# Tumor growth rate in pancreatic neuroendocrine tumor patients undergoing PRRT with ^177^Lu-DOTATATE

**DOI:** 10.1530/EC-21-0027

**Published:** 2021-03-19

**Authors:** Olof Joakim Pettersson, Katarzyna Fröss-Baron, Joakim Crona, Anders Sundin

**Affiliations:** 1 Radiology and Molecular Imaging, Uppsala University Hospital, Uppsala, Sweden; 2 Department of Surgical Sciences, Uppsala University, Uppsala, Sweden; 3 Department of Medical Sciences, Uppsala University, Uppsala, Sweden

**Keywords:** ^177^Lu-DOTATATE, pancreatic neuroendocrine tumors, peptide receptor radionuclide therapy, therapy monitoring, tumor growth rate

## Abstract

**Background:**

Monitoring of pancreatic neuroendocrine tumors (PanNET) undergoing peptide receptor radionuclide therapy (PRRT) with ^177^Lu-DOTATATE depends on changes in tumor size, which often occur late. Tumor growth rate (TGR) allows for quantitative assessment of the tumor kinetics expressed as %/month. We explored how TGR changes before and during/after PRRT and evaluated TGR as a biomarker for progression-free survival (PFS).

**Methods:**

In PanNET patients undergoing PRRT with ^177^Lu-DOTATATE from 2006 to 2018, contrast-enhanced CT or MRI was performed before and during the therapy. Patients with at least one hypervascular liver metastasis were included. TGR was calculated for the period preceding treatment and for two intervals during/after PRRT. Cox regression was used for the survival analysis.

**Results:**

Sixty-seven patients (43 men, 24 women), median age 60 years (range 29–77), median Ki-67 10% (range 1–30) were included. TGR before baseline (*n* = 57) (TGR_0_) was mean (s.d.) 6.0%/month (s.d. = 8.7). TGR at 4.5 months (*n* = 56) (TGR_4_) from baseline was −3.4 (s.d. = 4.2) %/month. TGR at 9.9 months (*n* = 57) (TGR_10_) from baseline was −3.0 (s.d. = 2.9) %/month. TGR_4_ and TGR_10_ were lower than TGR_0_ (TGR_4_ vs TGR_0_, *P* < 0.001 and TGR_10_ vs TGR_0_, *P* < 0.001). In the survival analysis, patients with TGR_10_ ≥ 0.5%/month (vs <0.5%/month) had shorter PFS (median = 16.0 months vs 31.5 months, hazard ratio 2.82; 95% CI 1.05–7.57, *P* = 0.040).

**Discussion:**

TGR in PanNET patients decreases considerably during PRRT with ^177^Lu-DOTATATE. TGR may be useful as a biomarker to identify patients with the shortest PFS.

## Introduction

Gastroenteropancreatic neuroendocrine tumors comprise a variety of tumors, originating from stem cells in the gastrointestinal canal and pancreatic islets. An incidence of approximately 5 per 100,000 person-years is often described (1, 2, 3, 4, 5). Pancreatic neuroendocrine tumors (PanNETs) are termed as either functioning or non-functioning based on the presence or absence of hormonal symptoms. The non-functioning tumors comprise up to 90% of all PanNETs while the functioning tumors, such as gastrinomas and insulinomas, are less common (1, 5, 6). Although most PanNETs are sporadic, some occur as part of an inherited syndrome, for example, MEN1, responsible for 20–30% of gastrinomas and less than 5% of insulinomas and rare functional tumors (6).

Patients with advanced disease, metastatic or inoperable locally invasive PanNETs often undergo systemic treatments such as chemotherapies or receive targeted molecular agents. Treatment with radiolabeled somatostatin analogs, often referred to as peptide receptor radionuclide therapy (PRRT), is usually recommended when other systemic therapies have failed (6, 7, 8).

Monitoring of NET patients undergoing systemic therapies relies on the response evaluation criteria in solid tumors (RECIST), currently RECIST 1.1 (9, 10). The RECIST 1.1 criteria are used to describe tumor response based on changes in the sum of the tumor diameters in morphological studies, using CT or MRI, and include guidelines for assessment of non-target lesions (9, 10).

For functional imaging of GEP-NETs, PET in combination with CT (PET/CT) with ^68^Ga-DOTA-somatostatin analogs (^68^Ga-DOTA-SSAs) is essential for lesion detection and staging in patients considered for PRRT. However, temporal changes in ^68^Ga-DOTA-SSA uptake have not yet been shown to reflect therapy outcome in patients undergoing PRRT (11, 12, 13).

Therapy monitoring based on tumor size changes is associated with several problems as well-differentiated NETs tend to stabilize or initially increase in size even when responding to the treatment (13, 14). Tumor shrinkage in patients during PRRT is usually a late event, as illustrated in a recent study on PanNET patients showing best response (RECIST 1.1) at median 14.8 months after start of ^177^Lu-DOTATATE therapy (15).

Attempts to mathematically discern tumor growth patterns has for long been in the scope of oncological research (16). Calculation of tumor growth rate (TGR) is based on changes in the sum of lesion diameters and allows for quantitative assessment of tumor kinetics (17, 18, 19, 20). Recent data indicate that TGR can be useful as an early biomarker in patients with small intestinal neuroendocrine tumors (SI-NET) and PanNETs undergoing a range of systemic therapies and watch and wait (18, 19, 20). Data also indicate that Ki-67 index at baseline may be associated with progression-free survival (PFS) (20). In the present study, we hypothesized that TGR in PanNET patients decreases during PRRT with ^177^Lu-DOTATATE. Moreover, we tested if TGR during therapy and Ki-67 at baseline could be associated with outcome parameters such as PFS.

The aim of the study was primarily to calculate TGR before and during/after PRRT with ^177^Lu-DOTATATE, to shed some light on the dynamics of TGR in this quite unique cohort of NET-patients, with PanNET patients exclusively that underwent PRRT. This is in contrast to the previous reports including various NET types undergoing different types of systemic therapies and watch and wait (18, 19, 20).

The second aim was to investigate if TGR could be used as a biomarker to differ non-responders from responders during treatment, preferably at an early stage of PRRT.

Thus, the present study did not aim to investigate whether or not TGR can replace the RECIST 1.1-criteria but, instead, if TGR may provide additional information to facilitate the radiological therapy monitoring.

## Materials and methods

### Patients and imaging

Patients were screened for inclusion using a previously described cohort of PanNET patients (*n* = 151) undergoing PRRT with ^177^Lu-DOTATATE (21). Inclusion criteria were at least one hypervascular liver metastasis at baseline on contrast-enhanced CT (CECT) of adequate technical quality. Portal-venous phase CECT was preferred. Contrast-enhanced MRI was used when CECT was not available. Patients harboring metastases with extensive calcifications or substantial necrosis were excluded, as this might affect lesion size in a manner unrelated to the effects of PRRT.

^177^Lu-DOTATATE, 7.4 GBq per cycle, was administered with 8–12 weeks intervals. PRRT was tailored according to kidney and bone marrow dosimetry to administer as many cycles as possible (22).

The sum of tumor diameters was obtained according to the modified RECIST 1.1 on CECT/MRI. We assessed the patients at four time points before/during treatment (please see ‘Calculation of TGR’). Data on Ki-67 and tumor grade at baseline were collected from the pathologists’ reports on biopsies of liver metastases or the primary PanNETs. Data on Chromogranin-A at baseline were obtained from the patients’ laboratory reports.

### Tumor measurements

In each patient, the sum of the longest diameters of three hypervascular liver lesions, two lymph node metastases and the primary tumor was measured. The RECIST 1.1 criteria were used to guide definition and subsequent selection of tumor lesions. For each patient, CECT/MRI examinations performed in similar contrast-enhancement phase were assessed at different points. When only an arterial phase CECT/MRI was available at baseline, this contrast-enhancement phase was consistently assessed in the CECT/MRI examinations performed at all the other time points.

The measurements of the sum of tumor diameters were based on the RECIST 1.1 criteria, with modifications of the number of lesions measured per organ (three instead of two per organ) and of the maximum interval between the baseline examination and the first therapy, 7 instead of 4 weeks.

### Outcome parameters

The patients underwent clinical, biochemical and radiological assessment at least every 3–6 months following PRRT. CECT/MRI was assessed according to RECIST 1.1 and the maximum percentage decrease/increase in the sum of tumor diameters (maximum two per organ, maximum five in total) compared to baseline was calculated to establish best response.

Partial response was defined as at least 30% reduction in the sum of tumor diameters and no new lesions. A 20% increase in the sum of tumor diameters and/or the appearance of new lesions were categorized as progressive disease and together with the patients’ clinical and biochemical data were allowed for establishing PFS. Overall survival (OS) was defined as the interval from initiation of therapy until death or the last known contact.

The RECIST 1.1 data were used to evaluate patient’s outcome (complete response, partial response, stable disease, and progressive disease).

Measurements based on modified RECIST 1.1 criteria described in the previous section ‘Tumor measurements’ were used to calculate TGR.

### Calculation of TGR

Calculation of TGR is based on the change of the sum of diameters for the target lesions and additionally incorporates time as a parameter, allowing for quantitative assessment of the tumor kinetics, expressed as percentage per month. Target lesions were identified according to the RECIST 1.1-criteria, with slight modifications, as described under ‘Tumor measurements’. TGR was calculated using a previously published formula (18, 20, 23, 24, 25):


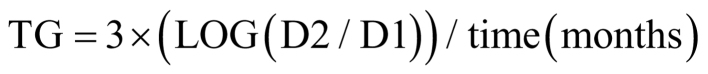



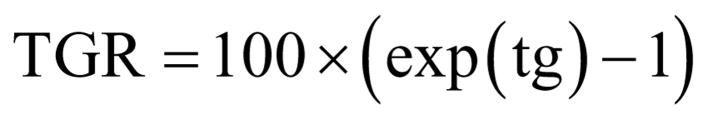


where TG is tumor growth, D1 is the sum of lesions at the earliest time point (e.g. the baseline study), D2 is the sum of lesions at the later point in time (e.g. a follow-up study). Time was calculated according to the formula: (late date − early date + 1)/30.44.

TGR was calculated for one interval before initiation of treatment and for two different intervals during PRRT. TGR was calculated between baseline and the most recent examination before baseline, within 12 months (TGR_0_) (18). TGR was also calculated between baseline and a study at about 4 months (TGR_4_) after baseline. Lastly, TGR was calculated between baseline and at about 10 months thereafter (TGR_10_). To extract the cohort of patients surviving and not progressing for at least 3 months from baseline, separate tumor growth rates were calculated after the 3-months landmark had been applied. TGR at the various intervals in the survival analysis were designated ‘TGR_0surv_’, ‘TGR_4surv_’ and ‘TGR_10surv_’ (please see also the ‘Statistical analysis’ section below).

### Statistical analysis

Kruskal–Wallis one-way ANOVA and Mann–Whitney *U*-test were carried out to compare TGR between groups. Shapiro–Wilk test was used to test for non-normal distribution vs normal distribution. Receiving operator characteristics (ROC) curves were used to search for cut-offs for TGR at different intervals. Univariate and multivariable Cox regression and Kaplan–Meier plots were used to test the biomarkers against the outcome parameters. To avoid the potential bias, ‘responders must live long enough for response to be observed and for TGR to be measured’, the landmark method was used in the survival analysis (18, 26). In line with a recent study, a 3-months landmark was set (18). Thus, patients dying or showing progressive disease at 3 months or earlier were excluded from the survival analysis. Kaplan–Meier estimates were also used to calculate PFS and OS in the cohort of included patients.

Two-sided tests with *P* < 0.05 were considered significant. In the Shapiro–Wilk test, *P* < 0.05 was used to test the hypothesis of non-normal distribution.

The statistical analyses were carried out using R 3.6.3 (R: A language and environment for statistical computing, R Foundation for Statistical Computing, Vienna, Austria; https://www.R-project.org/) with the packages ‘dplyr’, ‘ggfortify’, ‘ggplot2’, ‘ggsci’, ‘plotROC’, ‘ranger’ and ‘survival’.

## Results

### Baseline patients’ characteristics

**Figure 1:**
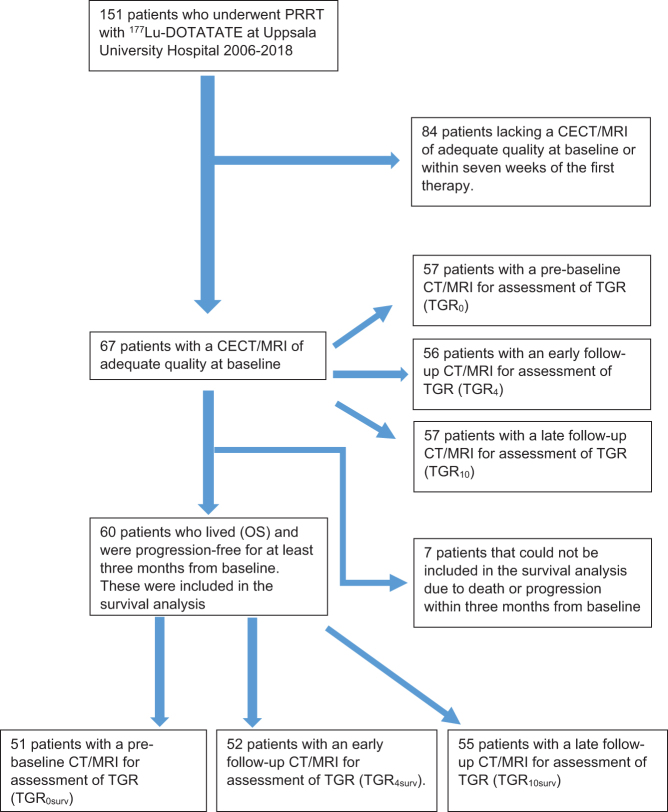
An overview of the number of PanNET patients included in each step. ‘Surv’ in the subscript denotes patients included in the survival analysis.

Out of the 151 patients who had undergone PRRT with ^177^Lu-DOTATATE at Uppsala University Hospital between 2006 and 2018, 67 patients had undergone CECT/MRI of adequate quality at baseline and could therefore be included (Fig. 1). The interval between the baseline examination and the first treatment was mean 1.49 weeks (range 0.13 to 6.70 weeks).

Baseline patient characteristics are described in Table 1.

**Table 1:** Baseline characteristics (*n* = 67).

	Value
Age (median, range)	60, 29–77
Gender (*n* = 67)	
Male	43
Female	24
Chromogranin-A, median^a^ (nmol/L)	18.25
Grade (based on Ki-67)	
Grade 1	7
Grade 2	46
Grade 3	5
Not specified	8
Ki-67%^b^ (median, range)	10, 1–30
Not specified	8
Metastases in or overgrowth to	
Liver	67
Lymph nodes	30
Bone	12
Adrenal glands	4
Peritoneum	2
Stomach	2
Ovaries	1
Spleen	3
Number of liver lesions (≥10 mm diameter)	
Only one lesion	1
Two lesions	7
Three or more lesions	59
Number of lymph nodes (≥15 mm short axis)	
One lymph node	15
Two or more lymph nodes	15
No enlarged lymph nodes	37
Primary tumor	
Not resected	47
Resected	11
Not delineated^c^	9
Type of tumor	
Non-functioning	41
Glucagonoma	5
Gastrinoma	4
Calcitoninoma	1
Insulinoma	1
5HIAA^d^	1
ACTH/G^e^	1
VIP^f^	1
Not specified	12

^a^Range 1.3 nmol/L to 168-fold the upper reference value (*n* = 61); ^b^biopsies were taken from a liver metastasis (*n* = 51) or the primary tumor (*n* = 8); ^c^the primary tumor could not be identified on the baseline examination; ^d^5-hydroxyindolacetic acid; ^e^adrenocorticotropic hormone; ^f^vasoactive intestinal peptide.

### PRRT with 177Lu-DOTATATE

Most patients underwent PRRT with three (*n* = 12), four (*n* = 21) or five (*n* = 8) cycles. Eleven patients received six cycles, six patients were given seven cycles and one patient was administered ten cycles. Eight patients were treated with two PRRT cycles. Four patients had undergone PRRT ^177^Lu-DOTATATE previously while the current treatment was to be considered a ‘salvage therapy’.

Out of the 67 included patients, 33 had stable disease as their best response, 30 reached partial response and four had progressive disease. Using Kaplan–Meier estimates, PFS was calculated to median 31.5 months (95% CI 27.0, *n* = 62) and OS was median 42.3 months (95% CI 33.3, *n* = 67).

### Imaging before and during PRRT

Out of the 67 included patients, the majority (*n* = 63) were examined solely by CECT. When no CECT was available, a contrast-enhanced T_1_-weighted MRI in the portal-venous phase was used for the measurements of tumor diameters. Four patients underwent a contrast-enhanced T_1_-weighted MRI before the baseline examination, used to calculate TGR_0_. One out of these four patients was also examined by a contrast-enhanced T_1_-weighted MRI at baseline. Another one out of these four patients had a contrast-enhanced T_1_-weighted MRI at the 10 months follow-up study, used to calculate TGR_10_.

### TGR before and during PRRT

TGR_0_ was calculated between CT/MRI performed mean 4.00 months (range 1.22 to 11.63) before baseline and the baseline examination (*n* = 57), TGR_4_ was calculated between the baseline examination and a study at mean 4.47 months after baseline (range 2.10 to 6.47 months) (*n* =56) and TGR_10_ was calculated between the baseline examination and a study at mean 9.94 months (range 8.05 to 11.99 months) (*n* = 57) after baseline.

**Figure 2:**
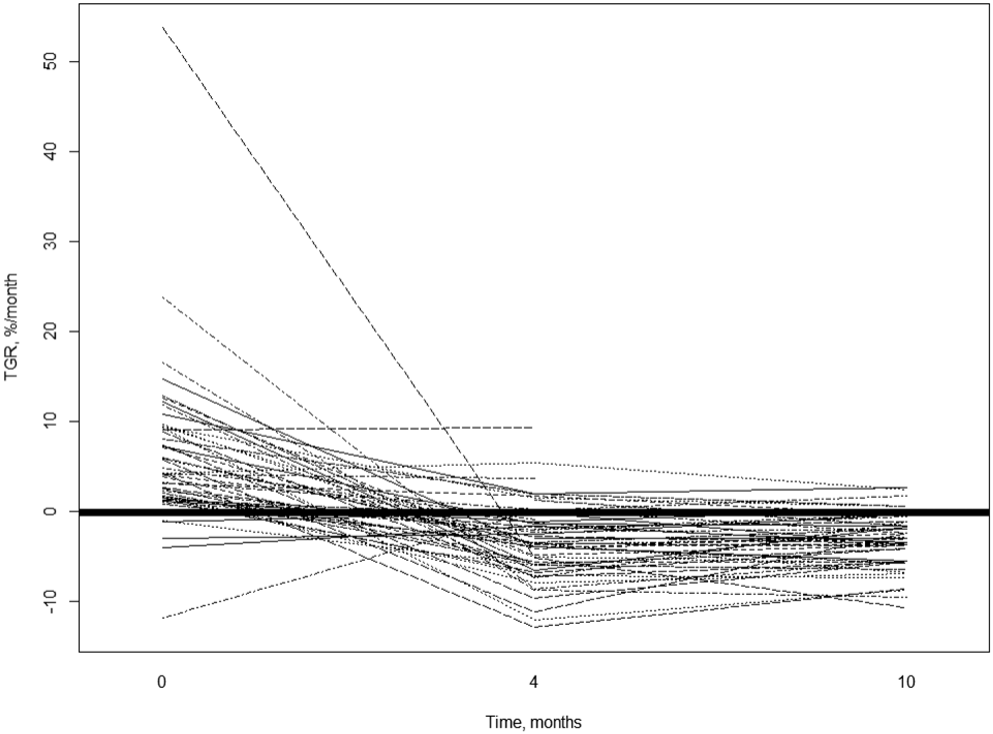
Spider plot of TGR between three different points in time. TGR was calculated from pre-baseline to baseline (0), from baseline to ~4 months (4) and from baseline to ~10 months (10). The solid, thick line marks mean TGR (−0.067%/month).

TGR for each individual patient is shown in Fig. 2. On the group level, TGR_0_ was mean (s.d.) 5.98 (8.66) %/month, TGR_4_ −3.36 (4.16) %/month and TGR_10_ −3.01 (2.92) %/month. Thus, TGR_0_ was 8.29%/month higher than TGR_4_ (median of difference by Mann–Whitney *U*-test, unpaired, 95% CI 6.61–10.25; *P* < 0.001). Compared to TGR_10_, TGR_0_ was 7.85%/month higher (95% CI 6.19–9.76, *P* < 0.001) (ANOVA *P* < 0.001 between the three groups). There was no significant difference between TGR_4_ and TGR_10_ (median of difference = 0.49, 95% CI −0.76 to 1.78, *P* = 0.41).

Shapiro–Wilk normality test indicated TGR_0_ deviated from a normal distribution (*P* < 0.001) in the present cohort while both TGR_4_ (*P* = 0.77) and TGR_10_ (*P* = 0.24) followed a normal distribution.

### Determination of a cut-off for TGR to predict progressive disease

ROC curves for TGR_0surv_, TGR_4surv_ and TGR_10surv_ plotted against the outcomes ‘progression at 12 months’ and ‘progression at 24 months’ were generated. TGR_0surv_ plotted against ‘progression at 12 months’ resulted in a ROC curve with an area under the curve (AUC) = 0.56 (95% CI 0.26–0.62) and TGR_0surv_ plotted against ‘progression at 24 months’ in a ROC curve with AUC = 0.57 (95% CI 0.24–0.58). TGR_4surv_ plotted against ‘progression at 12 months’ and ‘progression at 24 months’ resulted in ROC curves with slightly smaller AUCs (AUC 0.48; 95% CI 0.34–0.71 and AUC 0.42; 95% CI 0.42–0.75). TGR_10surv_ plotted against ‘progression at 12 months’ and ‘progression at 24 months’ resulted in ROC curves similar to the ones based on TGR_4surv_ with the same outcomes (data not shown).

As the resulting ROC curves were not useful to identify a cut-off to predict progressive disease, we set cut-offs for TGR_4surv_ and TGR_10surv_ based on earlier studies, although slightly modified (18, 20). Thus, the cut-off for TGR_4surv_ and TGR_10surv_ was set at 0.5%/month. The cut-off for TGR_0surv_ was set at 4%/month, as previously described (18, 25).

### Impact of TGR and other markers on patient outcome

**Figure 3:**
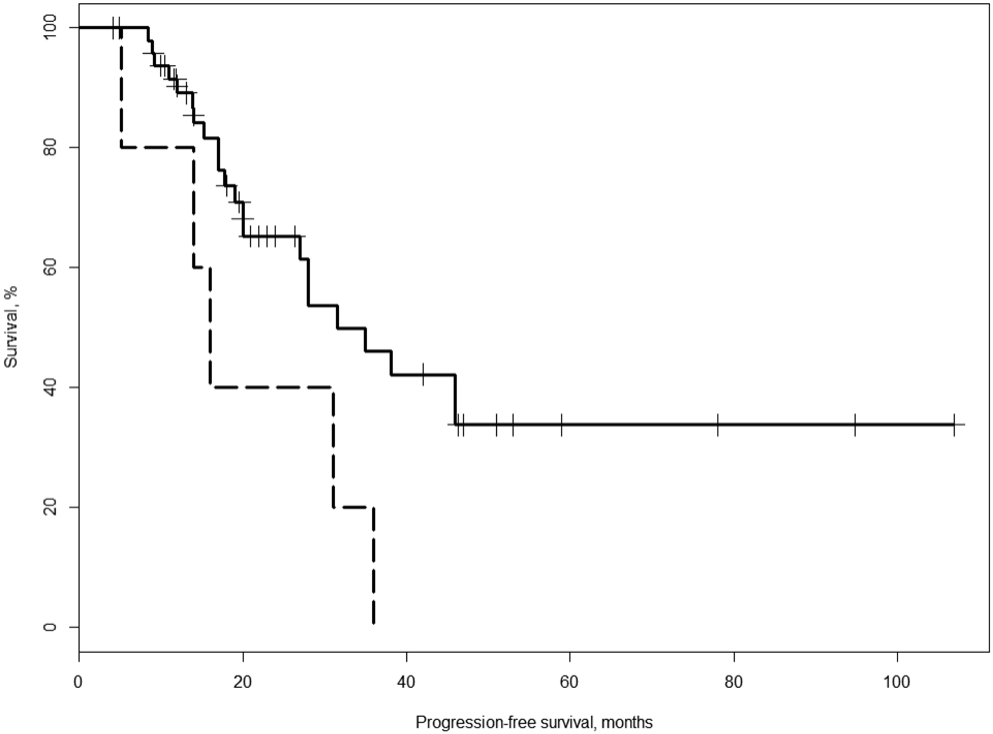
Kaplan–Meier plot of PFS. Patients with TGR_10surv_ < 0.5%/month (solid line, *n* = 50) and those with TGR ≥ 0.5%/month (dashed line, *n* = 5).

In the survival analysis, the patients (*n* = 55) with a CECT/MRI of adequate quality between baseline and 10 months were divided into two groups based on the 0.5%/month cut-off. Among these 55 patients, 50 patients had a TGR_10surv_ < 0.5%/month and 5 patients had a TGR_10surv_ ≥ 0.5%/month. The group with the TGR_10surv_ ≥ 0.5%/month had a shorter median PFS (median = 16.0 months) than the group with TGR_10surv_ < 0.5%/month (median = 31.5 months) (hazard ratio (HR) 2.82; 95% CI 1.05–7.57, *P* = 0.040; Fig. 3 and Table 2). TGR_10surv_ (≥0.5%/month vs <0.5) did not have a significant impact on OS (HR 2.145; 95% CI 0.783–5.879, *P* = 0.14).

**Table 2:** Univariate and multivariable Cox regression for PFS.

		Univariate analysis		Multivariable Cox regression	
		HR (95% CI)	*P*	HR (95% CI)	*P*
Age at first therapy (*n* = 60)	Continous variable	1.017 (0.983–1.052)	0.327	–	–
Gender (*n* = 60)	Male (vs female)	0.660 (0.314–1.386)	0.272	–	–
Ki-67 at baseline (*n* = 53)	<10 (vs ≥10)	0.392 (0.169–0.907)	0.029	0.383 (0.158–0.927)	0.033
Ki-67 at baseline (*n* = 53)	Continous variable	0.971 (0.979–1.082)	0.253	–	–
TGR_10surv_ (*n* = 55)	≥0.5%/month (vs < 0.5)	2.817 (1.048–7.57)	0.040	2.729 (0.986–7.546)	0.053
TGR_4surv_ (*n* = 52)	≥0.5%/month (vs <0.5)	2.008 (0.684–5.89)	0.204	–	–

In the multivariable model, only the parameters that had a significant impact on PFS in the univariable analysis were included.

PRRT, peptide receptor radionuclide therapy; TGR10surv and TGR4surv, tumor growth rates in the survival analysis.

**Figure 4:**
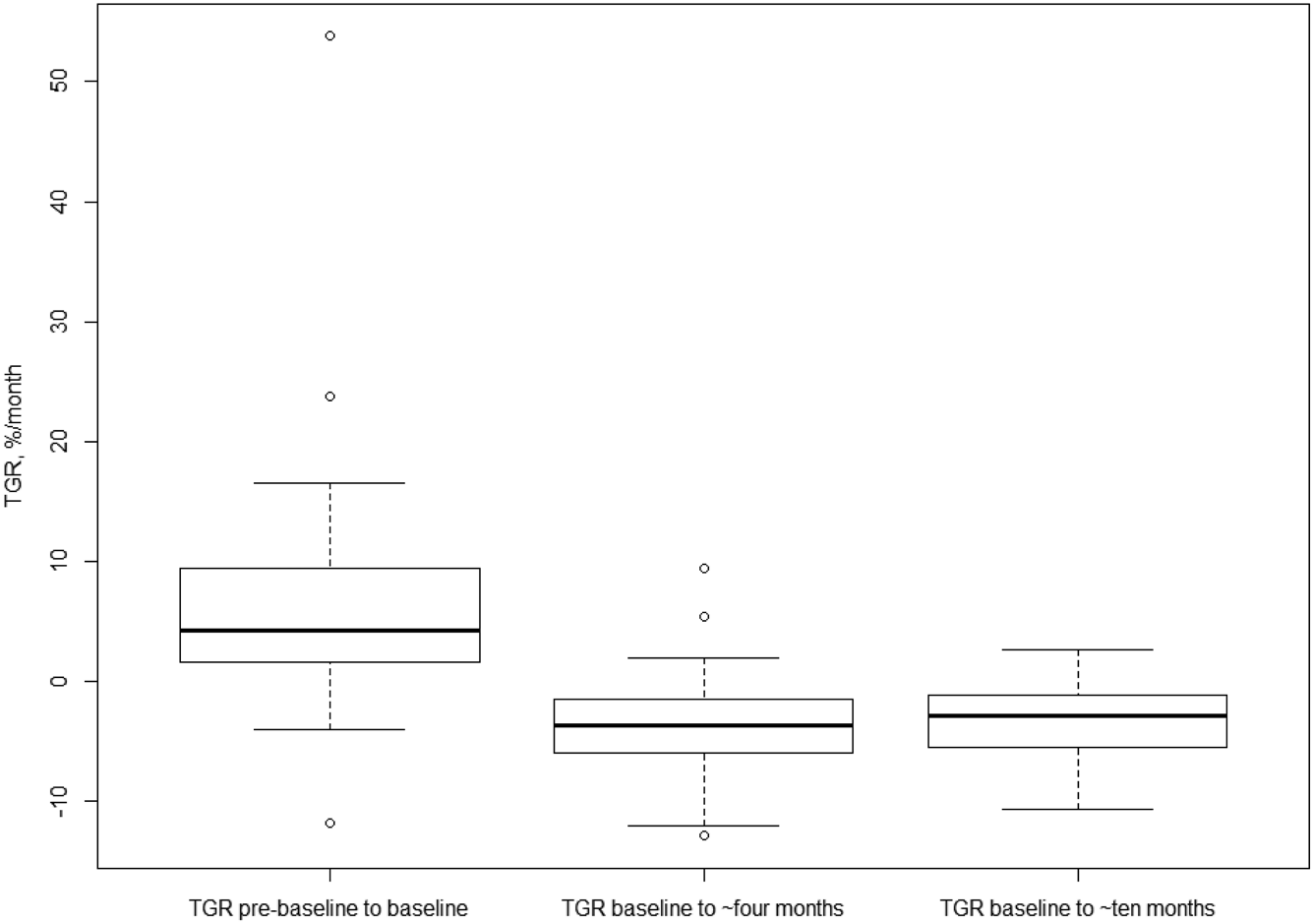
Boxplots of TGR_0_ (left), TGR_4_ (middle) and TGR_10_ (right) in the survival analysis. TGR_0surv_ was median 4.27 (range −11.83 to 53.79), TGR_4surv_ was −3.66 (−12.90 to 9.37) and TGR_10surv_ was −2.85 (−10.65 to 2.63).

When patients (*n* =52) were divided in two groups based on TGR_4surv_ with the 0.5%/month cut-off applied, there was no significant difference in PFS between the two groups (HR 2.01; 95% CI 0.68–5.89, *P* = 0.20, Table 2). Lastly, patients (*n* = 51) were divided in two groups based on TGR_0surv_ and the previously defined 4%/month cut-off. These two groups also did not have significantly different PFS (HR 0.70; 95% CI 0.32–1.54, *P* = 0.38). Also, neither TGR_4surv_ nor TGR_10surv_ had any significant impact on OS when the same cut-offs were used (data not shown). Thus, neither TGR_0surv_ nor TGR_4surv_ were included in the multivariable analysis. For an overview of TGR in the survival analysis, please see Fig. 4.

Apart from TGR, Ki-67 at baseline, the patients’ age at baseline and gender were tested in the univariate analysis (Table 2). Among these, ‘Ki-67 < 10%’ had an impact on PFS both in the univariable (HR 0.39; 95% CI 0.17–0.91, *P* = 0.029, *n* = 53) and in the multivariable analysis (HR 0.38; 95% CI 0.16–0.93, *P* = 0.033, *n* = 53). TGR_10surv_ (≥0.5%/month vs <0.5) did not have a significant impact on PFS (HR 2.73; 95% CI 0.99–7.55, *P* = 0.053) in the multivariable analysis. Please see Table 2 for an overview of the univariate and multivariable analyses.

## Discussion

In the present study investigating TGR in PanNET patients before and during PRRT with ^177^Lu-DOTATATE, those with a TGR of at least 0.5%/month from baseline to 10 months follow-up had a shorter median PFS (HR 2.82; 95% CI 1.05–7.57, *P* = 0.040; Fig. 3 and Table 2) compared to the rest of the cohort.

It may be noted that a few patients (*n* = 5) had a TGR of at least 0.5%/month and a median PFS of 16 months. This may be considered a rather long PFS for this quite high TGR. This indicates that it may be possible to have a positive TGR while demonstrating stable disease according to RECIST 1.1. This is also well in line with Kaplan–Meier models in recent reports (18, 20).

The in-treatment TGR in the present study was −3.18%/month, which is slightly lower than the mean TGR reported in SI-NET and PanNET patients undergoing a range of systemic therapies and watch and wait (18, 20). Thus, PRRT seems to lead to a more negative TGR in PanNETs than in other NET types. This is well in line with several studies reporting a high cytoreductive potential of PRRT in PanNET patients (7, 22, 27, 28). Moreover, the in-treatment cut-off for TGR in the present study was slightly lower than in previous reports (0.5%/month as opposed to 0.8%/month) (18, 19, 20). This is a consequence of the lower in-treatment TGR in the present study, again reflecting the high cytoreductive potential of PRRT with ^177^Lu-DOTATATE in PanNET patients.

As the number of patients with G1 and G3 tumors was fairly low, a subgroup analysis based on the WHO grading was not applicable in our material. Therefore, patients were dichotomized based on a Ki-67 index at 10% (15, 29). This turned out to be useful both in the univariable (HR 0.39; 95% CI 0.17–0.91, *P* = 0.029, *n* = 53) and in the multivariable (HR 0.38; 95% CI 0.16–0.93, *P* = 0.033, *n* = 53) analysis.

The findings that a positive TGR during systemic therapies is associated with a worse prognosis is well in line with recent retrospective studies of SI-NET and PanNET patients undergoing a range of systemic therapies and watch and wait (18, 19, 20). However, while these reports found both the pre-therapeutic TGR and the TGR during early therapy to be useful as biomarkers associated with outcome, we could only identify a useful cut-off for TGR between the baseline examination and about 10 months follow-up (18, 20). A notable difference between the previous studies and the present report is that we only included PanNET patients undergoing PRRT with ^177^Lu-DOTATATE.

As the present study was not able to generate ROC curves capable of identifying any cut-off useful in predicting progressive disease, our in-treatment cut-off was based on a previous study (18). By contrast, no useful cut-off for the pre-therapeutic TGR could be defined.

In therapy monitoring of PanNET patients undergoing PRRT with ^177^Lu-DOTATATE, the RECIST 1.1 criteria are used to measure changes in the sum of tumor diameters by CECT/MRI (10). As changes in tumor size tend to appear in late stages of the therapy, or even after finishing PRRT, there is a need for more precise methods to differentiate responders from non-responders earlier (13, 15, 30). Such methods could be a complement to the RECIST 1.1 criteria to identify the patients with a less favorable prognosis who need to be followed more closely vs those for whom these intervals may be longer. The present study reports a substantial reduction in TGR in PanNET patients during PRRT and we propose that TGR may be associated with PFS.

Among weaknesses in our work are that it is a single-center study, retrospectively evaluating a cohort of PanNET patients out of which more than 50% had to be excluded, mainly because the CT examination protocols were inconsistent and, therefore, did not allow for repeated comparisons over time in the same contrast-enhancement phase. These technical discrepancies between examinations were largely because patients underwent CECT, and in some cases MRI, at many different centers with local variations in their CT/MRI protocols.

In retrospective studies, it is often found that the technical quality of many CT examinations is suboptimal. Less than perfect examinations are usually sufficient for reading in the clinical context, but in a research study, a more homogenous material is advantageous and for this reason a quite substantial part of the cohort was excluded.

For similar reasons, patients harboring tumors with extended necrosis and/or calcifications were excluded. The motive for this was that tumor homogeneity, that is, patients with morphologically similar reasons, was considered important for less-biased testing of TGR before and during PRRT.

Given that TGR can be established as a tool to provide reliable information for therapy monitoring, future studies need to be extended to also include patients with heterogenous tumors.

Strengths in our study include that the present cohort is homogenous as it only includes patients with PanNETs. Also, all patients underwent the same treatment, receiving PRRT with ^177^Lu-DOTATATE, rather than a group of patients with a mixture of different NET types undergoing different therapies and watch and wait, as in recent studies evaluating TGR in GEP-NET patients (18, 19, 20).

Calculations on TGR were based on measurements of lesions by merely one observer. The interval between the first PRRT and the baseline examination was often longer than the 4 weeks stipulated by RECIST 1.1. The maximum interval in the present study was extended and set at 7 weeks. Also, there were rather wide variations for the interval between baseline examination and the first follow-up.

Although an increasing number of studies suggest TGR as a promising tool to monitor systemic therapies, its inherent weaknesses must be considered, such as the difficulties in handling complete responses. Moreover, neither the appearance of new lesions nor the assessment of non-target lesions is accounted for TGR. Thus, TGR in its present form needs to be used in conjunction with RECIST 1.1 and, unlike RECIST 1.1, at this point should not be considered an independent system for therapy monitoring.

In the clinical situation, TGR may be useful since a proper RECIST 1.1 evaluation in many departments is difficult to fit into the daily radiological routine. When RECIST 1.1 evaluations are nevertheless performed, they are frequently based on comparison with the previous examination and not executed ‘by the book’ using the baseline study (for PR) or nadir examination (for PD). The oncologist is, therefore, often required to keep track of the percentage changes and, thus, the intervals between examinations to assess the aggressiveness of the tumor. Here, TGR could be useful as it provides the percentage changes in tumor diameters per month, which can be readily calculated from two sequential examinations. Also, this needs to be assessed in a larger cohort in a prospective manner.

In conclusion, in PanNET patients undergoing PRRT with ^177^Lu-DOTATATE, TGR may decrease considerably during treatment. We also propose that TGR between baseline and 10-months follow-up should be further evaluated as a prognostic factor and that these findings should be investigated prospectively in a larger study. This also warrants future separate evaluations in patients with other NET types and undergoing different systemic therapies.

## Declaration of interest

Anders Sundin had lecture honoraria from Ipsen and from Advanced Accelerator Applications for external imaging expert work. Joakim Crona received lecture honoraria from Novartis and educational honoraria from NET Connect (funded by Ipsen). The remaining authors have no conflict of interest to declare.

## Funding

Joakim Crona received funding from The Swedish Cancer Society (Cancerfonden) through a Junior Clinical Investigator Award. Apart from this, the study received no external funding.

## Author contribution statement

Olof Pettersson and Anders Sundin designed the study and wrote the manuscript. Katarzyna Fröss-Baron and Anders Sundin provided the patients for the study. Olof Pettersson, Katarzyna Fröss-Baron and Anders Sundin collected and assembled data. All the authors took part in data analysis, interpretation and the final approval of the manuscript.

## Statement of ethics

The study was approved by the Local Ethics Committee for Human Research in Uppsala, Sweden (reference number: 2010/177 with later amendments) and all patients provided written informed consent. The declaration of Helsinki was followed.
